# PCDH18 is frequently inactivated by promoter methylation in colorectal cancer

**DOI:** 10.1038/s41598-017-03133-w

**Published:** 2017-06-06

**Authors:** Dan Zhou, Weiwei Tang, Guoqiang Su, Mingquan Cai, Han-Xiang An, Yun Zhang

**Affiliations:** 1 Key Laboratory of Design and Assembly of Functional Nanostructures, Fujian Provincial Key Laboratory of Nanomaterials, Fujian Institute of Research on the Structure of Matter, Chinese Academy of Sciences, Fujian, China; 2 Department of Medical Oncology, Xiamen Cancer Hospital, The First Affiliated Hospital of Xiamen University, Xiamen, Fujian China; 3 Xiamen Institute of Rare Earth Materials, Chinese Academy of Sciences, Xiamen, Fujian China; 4 Department of Gastrointestinal surgery, Xiamen Cancer Hospital, The First Affiliated Hospital of Xiamen University, Xiamen, Fujian China

## Abstract

Protocadherin18 (PCDH18) was found to be preferentially methylated and inactivated in colorectal cancer (CRC) using bioinformatics tools. However, its biologic role in tumorgenesis remains unclear. Herein, we aimed to elucidate its epigenetic regulation and biological functions in CRC. The methylation status of PCDH18 was significant higher in CRC tissues than in adjacent non-tumor tissues (median, 15.17% vs. median, 0.4438%). Expression level of PCDH18 was significantly lower in primary CRCs than in nonmalignant tissues. Importantly, methylation status of PCDH18 in cell-free DNA of CRC patients was also significantly higher than in healthy subjects. PCDH18 was readily expressed in NCM460 cells, but downregulated in 100% (4/4) of CRC cell lines by promoter methylation, despite its expression could be restored through demethylation treatment. Overexpression of PCDH18 suppressed CRC cell viability, colony formation and migration. Meanwhile, the depletion of PCDH18 by siRNA in NCM460 cells enhanced the colonogenicity and migration ability and promoted β-catenin nuclear accumulation, whereas it inhibited cell cycle arrest. These effects were associated with upregulation of phospho-GSK-3β and cyclin D1, and downregulation of caspase3 and p21. Our results suggested that PCDH18 was a putative tumor suppressor with epigenetic silencing in CRC and a potential biomarker for CRC diagnosis.

## Introduction

Colorectal cancer (CRC) is ranked as the third most common cancer in males and the second in females worldwide with roughly 1.40 million new cases diagnosed in 2012 (9.7% of all cancers)^1^. It is a heterogeneous disease that involves a multistep process whereby normal epithelium transforms into invasive cancer in inherited, familiar and sporadic forms^2^. Although the widespread use of invasive colonoscopy and innovative treatments has improved clinical outcomes and the overall survival rates of CRC patients, CRC remains the leading cause of cancer-related death^3^. The molecular carcinogenesis of CRC has not yet been fully clarified, but CRC appears to be driven by the accumulation of genetic and epigenetic alternations in tumor-suppressor genes (TSG) and oncogenes^4^.

Epigenetic alternations such as DNA methylation are the most common molecular alterations involved in CRC and are regarded as early events that contribute to tumor development^5^. DNA methylation associated with TSG silencing is believed to serve as a molecular biomarker for early diagnosis of CRC^6^. The overall survival rate of CRC patients is mainly associated with the tumor stage at the time of diagnosis. Traditional diagnostic factors are clinically useful but are lacking in accuracy (i.e., tumor sizes, histological grades and her2/neu overexpression)^7^. Thus, the identification of novel methylated genes may help better diagnose and predict the developing process of CRC. Epigenetic profiling based on bioinformatics analysis has often been carried out to identify potential TSG.

Protocadherins are a large subfamily of non-classical calcium-dependent adherin molecules. It has been reported that their main function is not only cell adhesion but also tightly linked to several major signaling pathways, including the Wnt/β-catenin signaling pathway^8^. Protocadherin 18 (PCDH18) is located on the chromosome 4q31 in humans and belongs to the protocadherins subfamily^9^. PCDH18 protein contains 6 extracellular cadherin repeats, a transmembrane domain and a particular cytoplasmic tail^10, 11^. It has been shown that PCDH18 is expressed in brain, heart, kindey, lung and trachea^12^. Some PCDHs including PCDH8, PCDH17 and PCDH20 were reported to be frequently silenced mainly in breast, prostatic, lung and digestive carcinomas via aberrant promoter methylation, indicating that PCDHs may function as TSGs^13–15^. In addition, another studies suggested that decreased expression of PCDHs (e.g. PCDH9, PCDH10 and PCDH20) facilitated epithelial-mesenchymal transition and migration through the Wnt/β-catenin signaling pathway, revealing that PCDHs may protect against malignant transformation^16, 17^. Although PCDH18 is in the same subgroup as PCDH8, PCDH17 and PCDH20 and involves in cell adhesion, behavior and migration during embryogenesis^18^, the precise role of PCDH18 in colorectal carcinogenesis remains unknown.

In this study, we identified that PCDH18 was preferentially hypermethylated in colorectal cancer using bioinformatics analysis. This was followed by a clinical validation study with multiple patient tissue and plasma samples that ultimately led to confirmation of PCDH18 as a potential biomarker for CRC diagnosis. Furthermore, we investigated the epigenetic regulation, biological function and molecular pathway of PCDH18 in CRC.

## Results

### Promoter of PCDH18 was hypermethylated in primary colorectal tissues, plasma and cell lines

**Figure 1:**
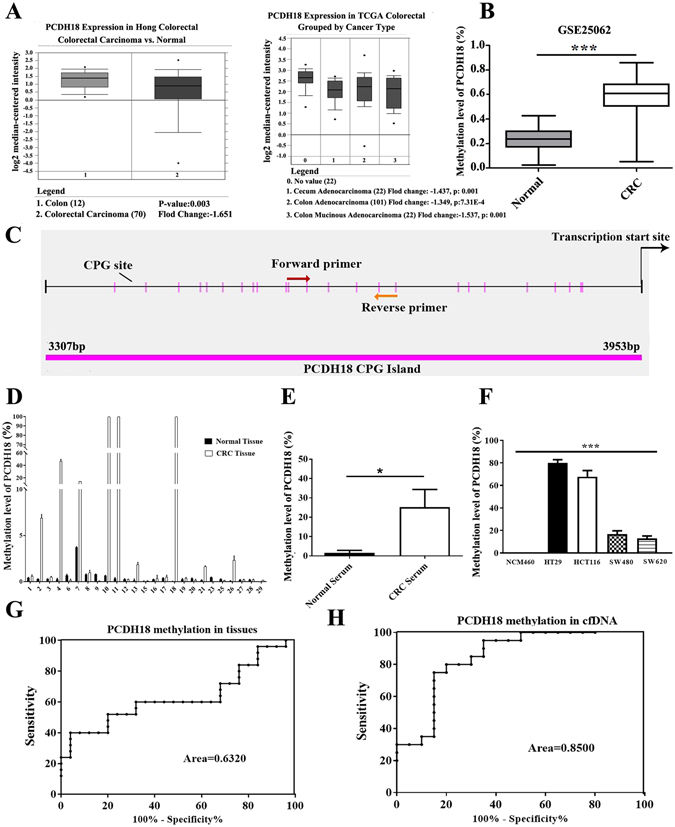
(**A**) Expression level of PCDH18 was significantly downregulated in colorectal cancer (CRC) tissues compared to normal controls in Hong Colorectal dataset and The Cancer Genome Altas expression array dataset from Oncomine. (**B**) A significant difference of PCDH18 methylation status was observed between 125 CRC tissues and 29 adjacent normal tissues in GSE25062 dataset. (**C**) Schematic structure of the PCDH18 promoter CpG islands, CpG sites (short vertical lines) and methylation specific PCR (MSP) region indicated (from forward primer to reverse primer). The transcription start site was showed by a curved arrow. (**D**) Methylation percentage of PCDH18 in 29 paired CRC tissues and normal tissues was detected by MSP. (**E**) Methylation percentage of PCDH18 in plasma from 20 CRC cases and 20 healthy subjects was examined by MSP. (**F**) Methylation percentage of PCDH18 in CRC cell lines (HT29, HCT116, SW480 and SW620) and normal colonic NCM460 cells were detected by MSP. (**G**) Receiver operating characteristic (ROC) curves showed the performance of PCDH18 methylation status from CRC tissues in predicting CRC. (**H**) ROC curves showed the performance of PCDH18 methylation status from CRC plasma in predicting CRC. Sensitivity means true-positive rate. Specificity means false-positive rate. Results were shown with means ± SD. *p < 0.05, ***p < 0.001.

In an attempt to identify PCDH18 involved in colorectal carcinogenesis, the Cancer Genome Altas (TCGA) expression array dataset of 145 CRC tissue samples and 22 paired normal samples as well as Hong colorectal dataset of 70 CRC tissue samples and 12 normal samples were obtained from Oncomine database for comparative genome-wide analyses of PCDH18 expression. Analysis of these data showed that PCDH18 expression was significantly down-regulated in CRC tissues compared with normal controls (Fig. 1A). Furthermore, we analyzed the methylation level of PCDH18 using R statistical software between 125 CRC tissues and 29 adjacent normal controls in GSE25062 dataset obtained from Gene Expression Omnibus database. We found that the methylation level of PCDH18 was significantly higher in CRC tissues than in adjacent normal controls (Fig. 1B). These data warrant further validation to demonstrate the accurate role of PCDH18 methylation with transcriptional silencing as a clinical useful biomarker for diagnosis of CRC patients.

To substantiate the potential role of PCDH18 in CRC, we first examined the methylation status of PCDH18 in 29 primary CRC tissue samples and paired normal tissues focusing on the 11–16 CpG sites in CpG island from 3307 to 3953 bp (Fig. 1C). As shown in Fig. 1D, PCDH18 hypermethylation was frequent in primary CRC tissues (25/29, 86.2%), and there were statistically significant differences in the methylation status of PCDH18 between CRC tissues (median, 15.17%, range, 0.0285–100%) and normal controls (median, 0.4438%, range, 0.0–3.712%)(Table 1). Next, we detected the methylation status of circulating cell-free (cfDNA) from 20 CRC patients and 20 healthy subjects by qMSP. We found the methylation level of PCDH18 was observably higher in CRC cfDNA (median, 25.17%, range, 0.0237–100%) than in normal controls (median, 1.655%, range, 0.0–23.67%) (Fig. 1E and Table 1). Subsequently, we detected the methylation status of PCDH18 in four CRC cell lines (HT29, HCT116, SW480 and SW620) and colonic NCM460 cells. PCDH18 gene promoter was shown to be ubiquitously hypermethylated in a panel of four CRC cell lines, especially in HT29 and HCT116 cells, whereas NCM460 cells showed an unmethylated status (Fig. 1F). The results were then verified by bisulfate sequencing PCR (BSP) (Supplementary Fig. 1). In addition, the evaluation of PCDH18 methylation levels in tissues and cfDNA circulating in plasma for CRC diagnosis using the receiver-operating characteristic (ROC) curves were shown in Fig. 1G and H. The analysis of PCDH18 methylation levels for the whole range of sensitivities and specificities using the area (0.6320 for tissues and 0.8500 for cfDNA) under ROC curves suggested that PCDH18 methylation levels could be useful molecular markers for CRC diagnosis. Collectively, these data indicated that PCDH18 was markedly hypermethylated in CRC.

**Table 1:** Methylation status (%) of PCDH18 in tissues and plasma.

	Tumor tissue study					Plasma study				
	n	CRC cases	n	Corresponding normal tissue	p	n	CRC cases	n	healthy controls	p
Total	25	15.17(0.0285–100)	25	0.4438(0.0–3.712)	0.0091	20	25.17(0.0237–100)	20	1.655(0.0–23.67)	0.0202

### Association between clinicopathologic characteristics and PCDH18 methylation status

Subsequently, the association between PCDH18 promoter methylation and clinicopathological characterictics was analyzed in 25 tissue specimens and 20 cfDNA from CRC patients. As shown in Table 2, there was no correlation between PCDH18 hypermethylation status and clinicopathological characterictics such as age, sex, tumor stage, or tumor location in 25 tissue specimens. However, in 20 plasma specimens, PCDH18 methylation status tended to be remarkably higher in age ≥ 55 group (median, 35.86%, range, 0.0237–100%) than age < 55 group (median, 0.218%, range, 0.0432–0.61%) (p = 0.0183 < 0.05).

**Table 2:** Association between clinicopathologic characteristics and PCDH18 methylation status.

Variables	Tissue			Plasma		
	n	Methylation status (%)	P	n	Methylation status (%)	P
**Age**						
≥55	13	17.02(0.06415–100.0)	0.7253	14	35.86(0.0237–100.0)	0.0183
<55	12	13.17(0,02850–100.0)		6	0.218(0.0432–0.61)	
**Sex**						
Male	14	18.48(0.07256–100)	0.9345	10	23.30(0.0432–100.0)	0.8495
Female	11	10.96(0.02805–100)		10	27.04(0.02368–100.0)	
**Stage**						
I + II	9	22.85(0.1724–100.0)	0.1752	5	20.21(0.05660–100.0)	0.2433
III + IV	14	12.23(0.02850–100.0)		12	30.99(0.09486–100.0)	
**Tumor location**						
Colon	12	18.22(0.06415–100.0)	0.8065	9	34.27(0.04320–100.0)	0.6201
Rectum	13	12.35(0.02850–100.0)		11	17.73(0.02368–100.0)	

### PCDH18 was epigenetically silenced in CRC tissues and cell lines

**Figure 2:**
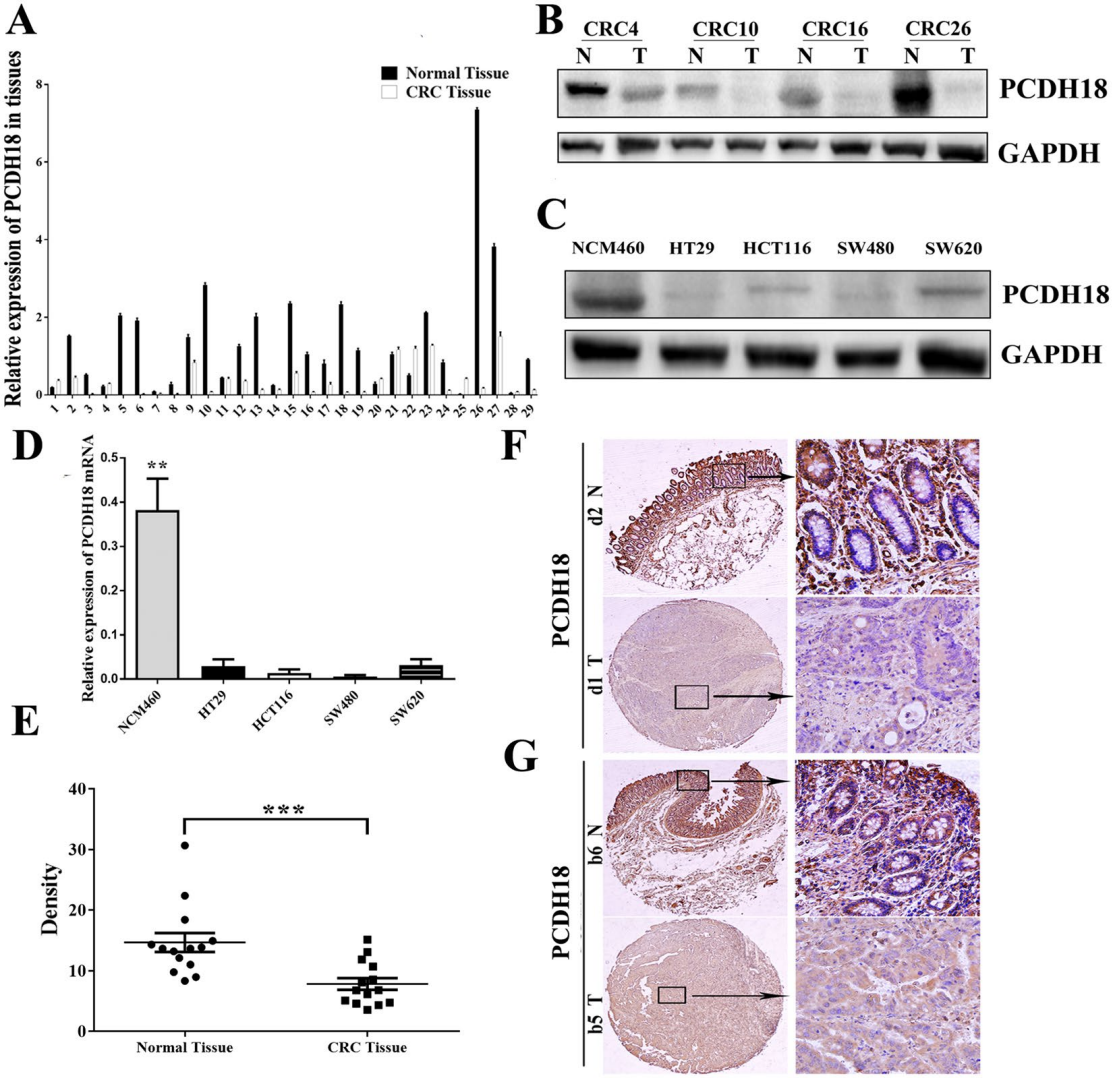
PCDH18 was epigenetically silenced in colorectal cancer tissues and cell lines. (**A**) PCDH18 mRNA expression levels in 29 paired CRC tissues and paired normal tissues were detected using quantitative PCR (qPCR), relative to the value of GAPDH in each sample. (**B**) ROC curves showed the performance of PCDH18 expression levels from CRC tissues in predicting CRC. (**C**) Representative Western blot image for protein expression level of PCDH18 in CRC tissues and paired normal tissues. (**D**) Western blot image for protein expression level of PCDH18 in CRC cell lines (HT29, HCT116, SW480 and SW620) and normal colonic NCM460 cells. (**E**) PCDH18 mRNA expression levels in CRC cell lines (HT29, HCT116, SW480 and SW620) and normal colorectal epithelial NCM460 cells were detected using qPCR, relative to the value of GAPDH in each sample. (**F**) Representative images of PCDH18 staining in CRC tissues and paired normal tissues. Normal tissues showed strong positive staining, whereas CRC tissue displayed weak staining. The right panel (magnification x400) was magnification of where inset in the left tissue array (magnification x100). (**G**) A statistically significant difference was detected between the mean density of CRC tissues and that of paired normal tissues in tissue microarray. Results were shown with means ± SD. **p < 0.01***p < 0.001.

To clarify whether DNA hypermethylation regulated the expression of PCDH18, we first compared PCDH18 expression level in 29 CRC tissues and their adjacent normal controls by qPCR and Western blot. As shown in Fig. 2A, PCDH18 was significantly downregulated in primary tumor tissues with PCDH18 hypermethylation compared to that in adjacent normal tissues. The area under the ROC curve (AUC) was 0.7753 (Fig. 2B). Western blot analyses showed that protein expression levels of PCDH18 were markedly repressed in 82.7% (24/29) of cases, especially in CRC tissues with hypermethylation of PCDH18 promoter (Fig. 2C). Next, we examined PCDH18 mRNA and protein expression levels in four CRC cell lines and the normal colonic NCM460 cells. As shown in Fig. 2D and E, the PCDH18 mRNA and protein expression level was significantly down-regulated or silenced in all four CRC cell lines (100%), but was readily detected in colonic NCM460 cells. Four CRC cell lines with PCDH18 hypermethylation revealed decreased or silenced PCDH18 expression. Subsequently, we investigated the protein expression of PCDH18 by immunohistochemical staining in 14 paired CRC and normal adjacent tissue from tissue microarray (TMA). While PCDH18 expression was weak in most of the CRC tissues (mean density, 7.839 ± 3.609), the majority of matched normal tissues showed much higher PCDH18 positive staining with an average density of 14.69 ± 5.888 (Fig. 2F and G). Collectively, these results demonstrated that DNA methylation was responsible for loss of PCDH18 expression.

### Demethylation agent 5-aza-2′-deoxycytidine (5-AZA) restored PCDH18 expression in CRC cell lines

**Figure 3:**
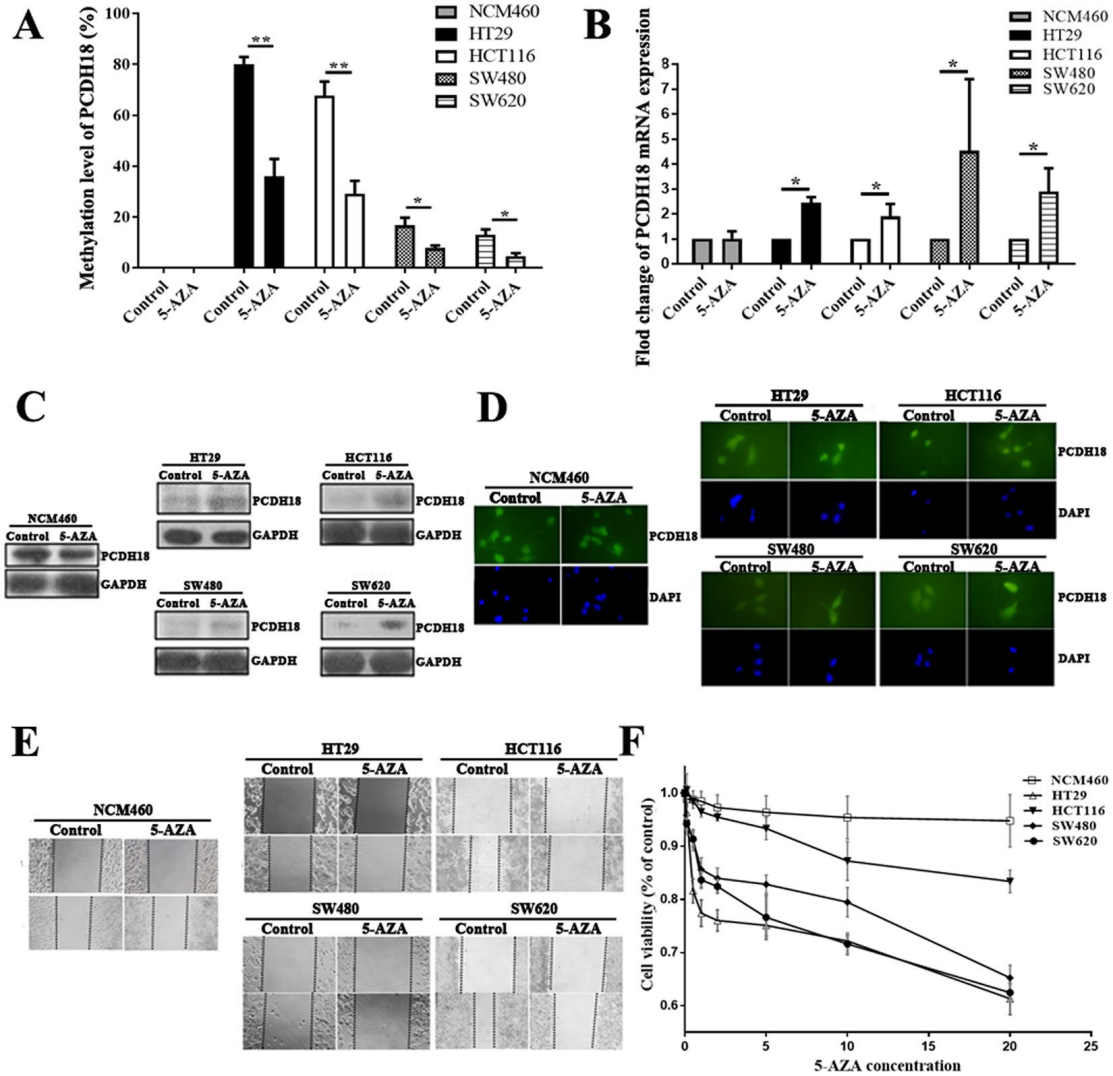
Demethylating agent 5-aza-2′-deoxycytidine (5-AZA) treatment induced PCDH18 expression and inhibited migration and growth in CRC cell lines. (**A**) PCDH18 methylation status after 5μm 5-AZA treatment in CRC cell lines (HT29, HCT116, SW480 and SW620) and normal colorectal NCM460 cells are measured by MSP. QPCR (**B**), Western blot (**C**) and Immunofluorescence staining (**D**) were used to examine mRNA and protein expression levels of PCDH18 after 5 μm 5-AZA treatment in CRC cell lines and NCM460 cells. (**E**) CRC cell lines treated with 5 μm 5-AZA were examined the ability of cell migration compared with NCM460 cells by Wound healing assays. (**F**) Growth curve of CRC cell lines and NCM460 cells treated with various concentrations of 5-AZA. A negative control treated with PBS (without 5-AZA) were included in each assay. Results were shown with means ± SD. *p < 0.05, **p < 0.01.

To further confirm the association between silencing and methylation status of PCDH18, we treated CRC cell lines (HT29, HCT116, SW480 and SW620) as well as the colonic NCM460 cells with demethylation agent 5-AZA. As expected, CRC cell lines treated with 5-AZA, which initially showed high level of PCDH18 methylation, was induced to re-express PCDH18 after 5-AZA treatment (Fig. 3A). QPCR and Western blot analyses showed that the ratio of PCDH18 to GAPDH mRNA and protein expression was increased by several fold in the 5-AZA-treated group compared to that in the control group (Fig. 3B and C). For immunofluorescence (IF) analyses, PCDH18 expression level in CRC cell lines exposed to the 5-AZA treatment was higher than control group (Fig. 3D). This increase was displayed in terms of total protein content with both cytoplasmic and nuclear locations in all CRC cell lines. In contrast, the methylation and expression level of PCDH18 did not change significantly after 5-AZA treatment in NCM460 cells, strongly indicating that CpG island hypermethylation was responsible for loss or decreased expression of PCDH18 in colorectal cancer.

To address whether PCDH18 might regulate CRC cells migration and proliferation, we initially investigated the effects of re-expression of PCDH18 after 5-AZA treatment on the migration of four CRC cell lines and colonic NCM460 cells using the monolayer scratch-healing assay. As shown in Fig. 3E, restoration of PCDH18 markedly slowed CRC cell lines migration at the edges of scratch wounds in monolayer of CRC cells. In addition, to evaluate re-expression of PCDH18 on the cell viability, four CRC cell lines and normal NCM460 cells were treated with various concentrations of 5-AZA and analyzed using CCK8 assay. A concentration inhibition of cell proliferation was observed in all CRC cell lines (Fig. 3F). However, 5-AZA treatment had no influence on the migration and growth of nonmalignant NCM460 cells. These data indicated that 5-AZA inhibited the proliferation and migration of CRC cells.

### Overexpression of PCDH18 suppressed cell migration and proliferation in CRC cell lines

**Figure 4:**
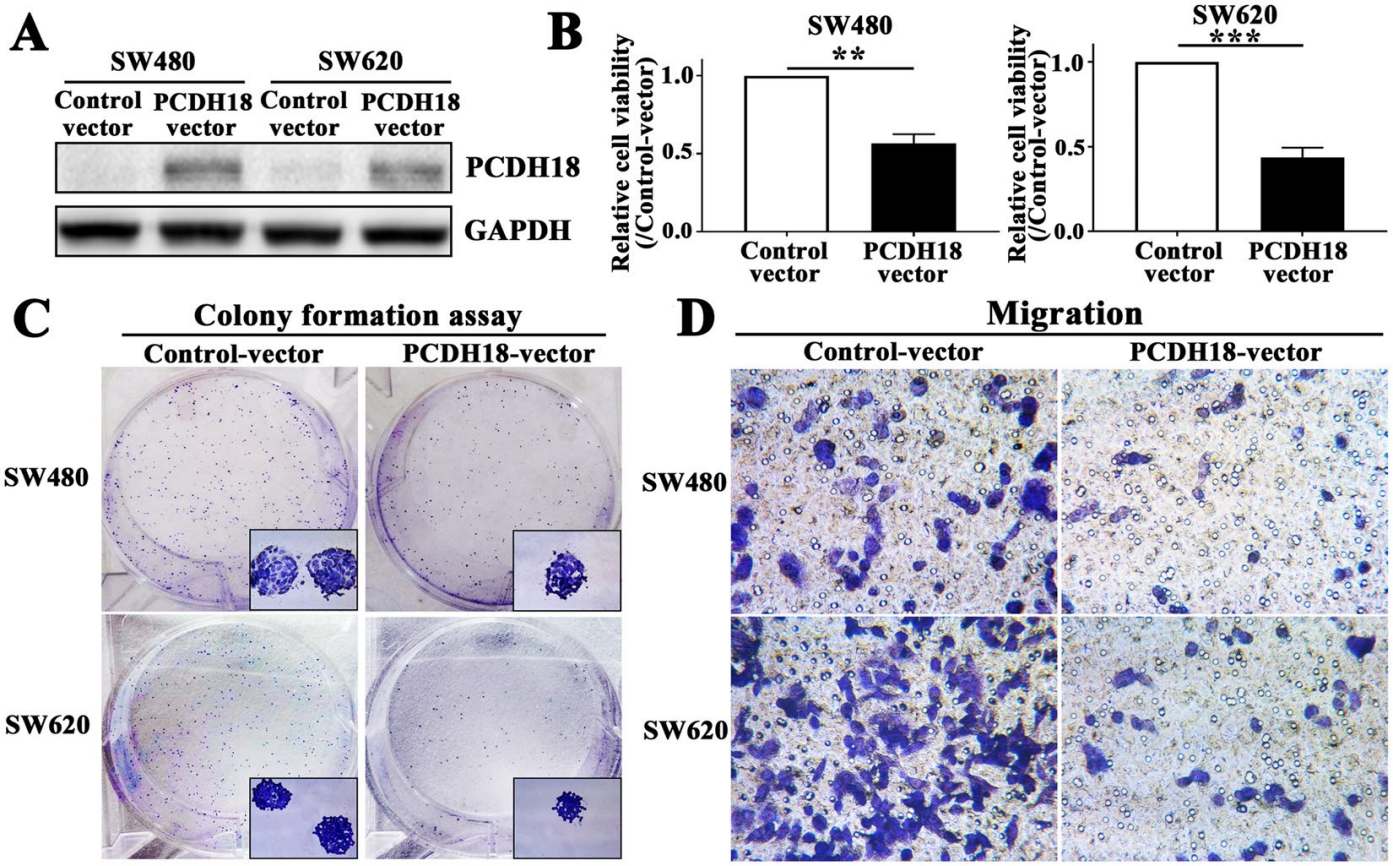
Overexpression of PCDH18 suppresses cell migration and proliferation in CRC cell lines. (**A**) Expression of PCDH18 in SW480 and SW620 cells after transfection with a PCDH18 expression vector was confirmed by Western blot. (**B**) PCDH18 expression significantly suppressed the number of viable cells of PCDH18-transfected SW480 and SW620 cells compared to that of control vector-transfected cells. (**C**) Representative images of colonies in PCDH18-transfecting as well as control vector-transfecting SW480 and SW620 cells. (**D**) Representative transwell assays in PCDH18-transfecting as well as control vector-transfecting SW480 and SW620 cells. Results were shown with means ± SD. *p < 0.05, **p < 0.01.

The above results, which showed epigenetic inactivation of PCDH18 in CRC but not in normal tissues, suggested a potential tumor suppressor role of PCDH18. To verify this hypothesis, we examined the growth inhibition effect by ectopic PCDH18 in SW480 and SW620 cells with suppressed PCDH18 expression. Overexpression of PCDH18 in transiently and stably transfected SW480 and SW620 cells was confirmed by Western blot (Fig. 4A). Ectopic expression of PCDH18 in these CRC cells induced a significant decrease in cell viability as detected by the CCK8 assay (Fig. 4B). Subsequently, results of colony formation assays showed that the number of SW480 and SW620 cell colonies significantly less as well as smaller in size than those of control vector-transfected cells (Fig. 4C). Next, a transwell assay was performed to further confirm the suppressive role of PCDH18 in CRC cell migration. As shown in Fig. 4D, the number of migrated cells was significantly decreased in PCDH18-overexpressing SW480 and SW620 cells. These results further suggested that PCDH18 negatively regulated CRC cells proliferation and migration.

### PCDH18 suppression promoted cell migration and proliferation in colonic NCM460 cells

**Figure 5:**
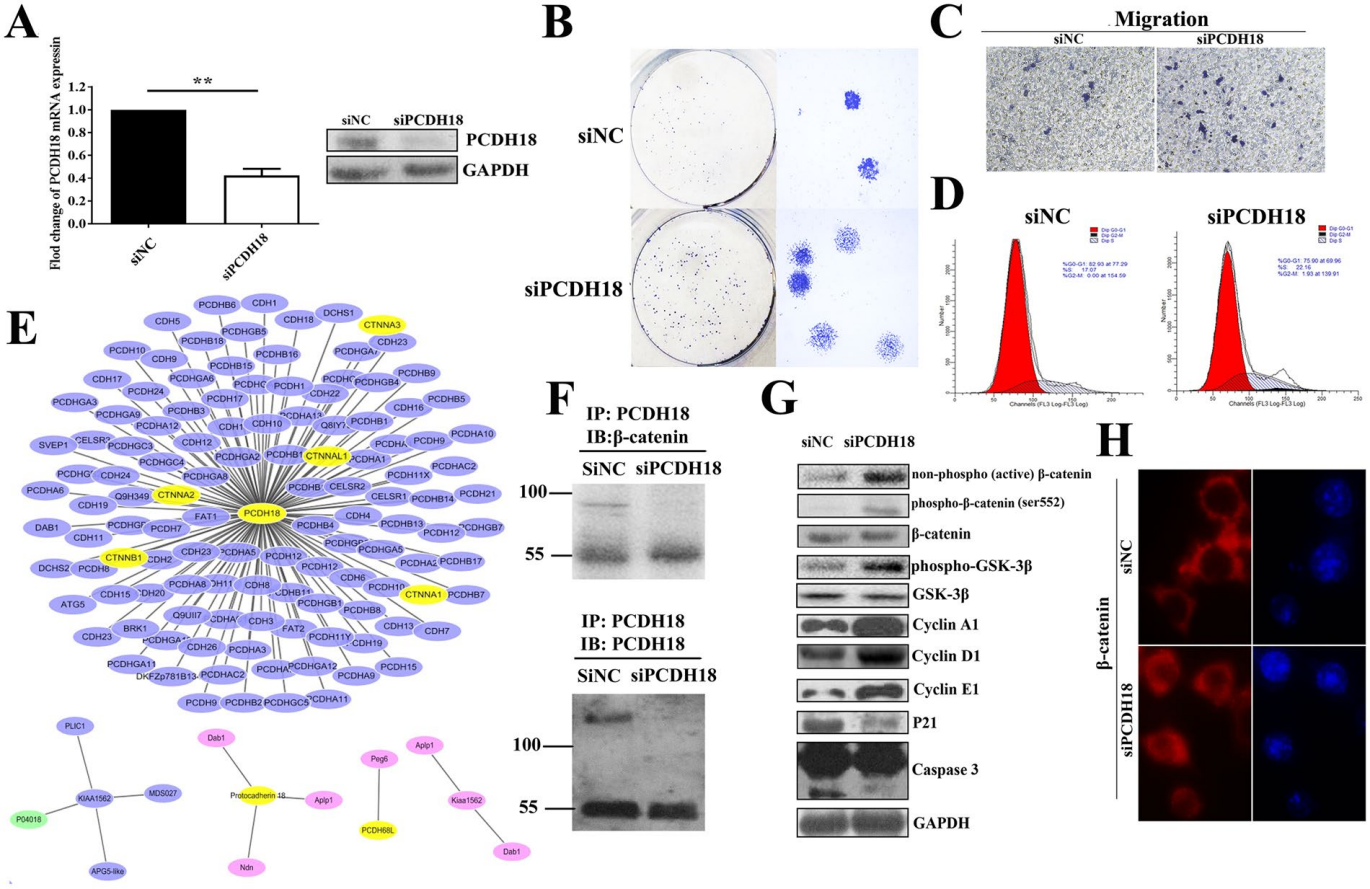
PCDH18 suppression promoted cell migration and proliferation in colonic NCM460 cells through the Wnt/β-catenin signaling pathway. (**A**) PCDH18 mRNA and protein expression was efficiently inhibited in NCM460 cells transfected with siPCDH18. GAPDH was used as an internal control. (**B**) Representative colony formation assays in NCM460 cells transfected with siRNA-PCDH18 (siPCDH18) and siRNA-negative control (siNC). (**C**) Representative transwell assays in NCM460 cells transfected with siPCDH18 and siNC. (**D**) Cell cycle distribution of NCM460 cells transfected with siPCDH18 and siNC was detected by flow cytometry analysis. (**E**) Molecular interaction networks involving PCDH18 were visualized by Cytoscape3.3.0 software from different species. Blue node represents gene from Homo species. Red node represents gene from Mus species. Green node represents gene from Bos taurus species. (**F**) Protein expression levels of nonphospho (active)-β-catenin, phospho-β-catenin, total-β-catenin, phospho-GSK-3β, GSK-3β, p21, cyclin A1, cyclin D1, cyclin E1 and caspase3 in NCM460 cells transfected with siPCDH18 and siNC. (**G**) Representative images of β-catenin staining in NCM460 cells transfected with siPCDH18 and siNC.

NCM460 cells had higher PCDH18 expression and lower migratory potential than CRC cell lines. To further validate the biological role of PCDH18, nonmalignant NCM460 cells were transfected with PCDH18-siRNA (siPCDH18) and negative control-siRNA. The suppression of PCDH18 mRNA and protein expression was demonstrated by qPCR and Western blot (Fig. 5A). To investigate the effect of PCDH18 on cell proliferation, colony formation assays were conducted. A sharp increase of colonies and colonies larger in size were observed in cells transfected with siPCDH18 in comparison with NCM460 cells transfected with negative control (Fig. 5B). Furthermore, to evaluate the effect of PCDH18 on cell migration, a transwell assay was carried out. The number of cells that migrated through the memberane into the lower chamber was significantly higher in cells transfected with siPCDH18 than in control cells (Fig. 5C). To explore whether PCDH17 influenced cell cycle, flow cytometry analysis was conducted. Cell cycle analysis showed G0/1 phase was decreased, whereas S and G2/M was increased in NCM460 cells transfected with siPCDH18 (Fig. 5D). Western blot further confirmed an upward trend in cyclin A1 and cyclin E1 and a downward trend in p21 protein expression in NCM460 cells transfected with siPCDH18. Meanwhile, expression level of cleaved caspase 3 was significantly lower than the control group (Fig. 5G). Taken together, these results suggested that sufficient PCDH18 levels were critical for maintaining the balance of migration and proliferation in normal colonic epithelial cells.

### PCDH18 regulated the Wnt/β-catenin signaling pathway

We next explored the molecular mechanisms underlying the regulation of cell migration and proliferation by PCDH18. Firstly, molecular interaction networks involving PCDH18 were visualized by Cytoscape3.3.0 software, and consist of 130 genes including CTNNB1 encoding β-catenin (Fig. 5E). Moreover, as previous reports suggested that certain PCDHs were capable of directly binding to β-catenin and antagonizing its activity^19^. Therefore, we further investigated whether PCDH18 negatively regulated the migration and proliferation of colonic cells by affecting the Wnt/β-catenin signaling pathway. Firstly, we analyzed co-immunoprecipitation of PCDH18 and β-catenin protein. As shown in Fig. 5F, immunoprecipitation of PCDH18 from lysates of NCM460 cells transfected with negative control coprecipitated β-catenin, although the positive band was weak. Subsequently, we examined downstream target gene of Wnt/β-catenin signaling pathway using Western blot. We found that cyclin D1 was significantly increased after PCDH18 suppression in NCM460 cells (Fig. 5G). Finally, we examined the protein level and localization of the β-catenin in siPCDH18-transfected NCM460 cells. As shown in Fig. 5G, levels of nonphospho (active)-β-catenin and phospho-β-catenin (ser552) markedly increased in PCDH18-suppressing NCM460 cells compared with negative control, accompanied by upregulated expression of phospho-GSK-3β. Meanwhile, no significant changes were observed in total β-catenin and GSK-3β protein levels. In addition, IF showed that suppression of PCDH18 in NCM460 cells resulted in significant nuclear accumulation of β-catenin (Fig. 5H). Collectively, these results suggested that Wnt/β-catenin signaling pathway played a critical role in PCDH18-mediated colonic cell migration and proliferation.

## Discussion

Colorectal cancer remains associated with significant mortality worldwide. It is a great challenge of basic and clinical research to explore precise biomarkers for diagnosis and therapy of colorectal cancer. In our present study, we have identified PCDH18 as a candidate biomarker for diagnosis of colorectal cancer and a candidate TSG for CRC. We demonstrated that PCDH18 was frequently down-regulated in CRC tumors and all CRC cell lines analyzed, whereas widely expressed in NCM460 cells and normal tissues. PCDH18 down-regulation in CRC cell lines was associated with its promoter methylation which could be induced by pharmacological demethylating agent. Subsequently, we clarified that overexpression of PCDH18 in CRC cell lines suppressed cell viability, colony formation and migration. Meanwhile, its depletion enhanced the migration and proliferation capability of normal colonic epithelial cell. Importantly, the Wnt/β-catenin signaling pathway was found to be involved in this process. To the best of our knowledge, this is the first report to describe the critical role of PCDH18 in colorectal cancer.

Detection of methylated cfDNA circulating in the plasma has been suggested to be a noninvasive biomarker for detecting the presence of various malignancies^20–23^. Several studies have described a high concordance between the epigenetic alterations detected in primary tumor specimens and in plasma or serum^24^. In this study, we found that PCDH18 promoter methylation was a common epigenetic event in CRCs, and that cancer-specific aberration could be frequently found in the circulation. We could detect PCDH18 hypermethylation in approximately 75% of cfDNAs extracted from CRC patients with high diagnostic value for CRC (AUC = 0.8500). We also showed that old age (≥55) which was a risk factor for CRC progression as bearing strong correlation with the PCDH18 methylation in cfDNA. The results of our study further suggest the possibility of using plasma biomarker assay for early detection strategies.

In this study, we have also observed that PCDH18 promoter methylation not only existed in CRC tissues but also in adjacent normal tissues, which may indicated that PCDH18 methylation was an essential trigger for CRC initiation. Furthermore, we found that the frequency of PCDH18 methylation in CRC tissues is not as high as that in CRC cell lines indicating that other mechanisms mediating transcription, including loss of heterozygosity and gene mutation, could further repress gene expression. Also, tissue samples contains various types of cell and each cell type may regulation PCDH18 expression with a specific mechanism in which promoter methylation may or may not be involved^25^.

In the current study, a concentration-dependent inhibition of proliferation of CRC cell lines (HT29, HCT116, SW480 and SW620) were identified following treatment with various concentrations of pharmacologic demethylating agent 5-AZA. Additionally, 5-AZA treatment resulted in the restoration of PCDH18 gene expression which is silenced by hypermethylation. The reason for the inhibition of proliferation of CRC cell lines might be the contribution of PCDH18 re-expression. It is also possible the cytotoxicity of 5-AZA leads to an anticancer influence. However, numerous studies reported that 5-AZA could not lead to the inhibition of cancer cell proliferation through an anticancer effect by exerting cytotoxity^26, 27^. Meanwhile, normal colorectal NCM460 cells were treated with 5-AZA using the same concentration, but no significant effect on cell proliferation was observed. Thus, the demethylating agent 5-AZA is a potential anticancer agent for effective management of colorectal carcinomas.

With regard to the underlying mechanism of PCDH18 in cancer pathogenesis, there is still little to no studies focusing on the potential role of PCDH18 in CRC. Similar to other protocadherin family members, the gene structure of PCDH18 is highly conserved and comprised of 4 exons^28^. A previous study revealed that overexpression of PCDH18 diminished cell migration and reduced cell protrusions^29^. Meanwhile, knock-down of PCDH18 using a morpholino oligonucleotide results in reduced cell adhesion. Recent studies demonstrated that some protocadherins act as tumor suppressors through interacting with Wnt/β-catenin signaling pathway, including PCDH8, PCDH10, PCDH17 and PCDH20^15, 30, 31^. Those results were complementary to ours and suggested that more genes in this family may be targets for inactivation during carcinogenesis^32^. In our study, PCDH18 was found to neutralize the Wnt/β-catenin signaling pathway in colonic NCM460 cells through inactivating GSK-3β. Interestingly, co-immunoprecipitation of PCDH18 with β-catenin was detected from lysates of NCM460 cells. This finding might be explained as follows: PCDH18 methylation resulted in its decreased expression and released β-catenin from PCDH18-β-catenin complex. Meanwhile, increased GSK-3β phosphorylation facilitated the stability of β-catenin and enables its nuclear transport. Further studies should be carried out to explore the exact downstream molecular mechanism activated by PCDH18.

MSP technique with SYBR green based quantitative PCR has been conducted to detect the methylation status of PCDH18 in this study. Unlike the classical qMSP using Taqman probes, the new method is a quantitative, reproducible and cheaper method, which uses methylation percentage to quantify methylated DNA^33^. Additionally, the products of the qMSP can be confirmed by sequencing^34^. Therefore, MSP technique with SYBR green based quantitative PCR is reliable and simple in large-scale epidemiologic studies.

In our study, we identified and characterized a potential biomarker, PCDH18, in colorectal cancer that involved in cell proliferation and migration through the Wnt/β-catenin signaling pathway. Our results will be useful for clarification of the relationship between PCDH18 methylation and its expression, thereby providing a candidate therapeutic target for the treatment of colorectal cancer. Nevertheless, sample size is a crucial factor to gain statistically significant results. The clinical samples and CRC cell lines employed in this study were relatively limited. Hence, large-scale studies should be performed to investigate the clinical significance of PCDH18 in CRC in the future.

## Materials and Methods

### Bioinformatics analysis from online databases

To determine the expression pattern of PCDH18 in colorectal cancer, the datasets in Oncomine database (https://www.oncomine.org) were analyzed. Briefly, PCDH18 gene was queried in the database and the results were filtered based on selecting colorectal cancer and colorectal cancer vs. Normal Analysis. Details of standardized normalization techniques are explained on the Oncomine.

Genome-scale DNA methylation array data of 125 colorectal tumor samples and 29 normal-adjacent colonic tissues samples was downloaded from the Gene Expression Omnibus database (GEO, http://www.ncbi.nlm.nih.gov/geo/, GSE25062^35^). The dataset was generated on the platform of Illumina Human Methylation 27 BeadChip which could interrogate 27,578 CpG sites. The methylation status of PCDH18 within individual samples was summarized using Biobase, GEOquery and limma on R statistical software.

### Specimens and Cell lines

Primary CRC and adjacent normal tissue specimens obtained during surgery from 29 CRC patients undergoing tumor resection at the First Affiliated Hospital of Xiamen University with histologically verified CRC between July 2013 and July 2016 were frozen immediately in liquid nitrogen and stored at −80 °C until required. Blood specimens from 20 CRC patients and 20 healthy subjects were drawn before therapeutic intervention. Plasma was collected from the 4 ml blood specimens after centrifugation at 1,600 × g for 10 min and 10,000 × g for 10 min and stored at −80 °C until processing for DNA extraction. All experimental protocols were approved by the Clinical Research Ethics Committee of the First Affiliated Hospital of Xiamen University. All methods were performed in accordance with relevant Guidelines and regulations. Written informed consent was obtained from all human participants after complete description of the study. Demographic, clinical and histopathological parameters of all these participants were shown in Supplementary Table. S1.

Four CRC cell lines (HT29, HCT116, SW480 and SW620) and an immortalized human colonic epithelial cell line (NCM460) were cultured in RPMI-1640 medium (Gibco cat#11875093) supplemented with 10% fetal bovine serum and incubated at 37 °C in a humidified atmosphere with 5% CO_2_. All the cell lines were obtained from the Cancer Center of Xiamen University (Xiamen, China). Cells were authenticated by short tandem repeat (STR) fingerprinting by Beijing Microread Genetics Company Limited recently.

### 5-Aza-2′-deoxycytidine (5-AZA) treatment

Four CRC cell lines and normal NCM460 cells were seeded in 96-well plates at a density of 5 × 10^3^ to a final volume of 200 μl and treated with various concentrations (0.1, 0.5, 1, 2, 5, 10, 20 μM) of 5-AZA (Sigma Aldrich cat#A3656) diluted in phosphate-buffered saline (PBS) (Gibco cat#10010023) for 48 h. A negative control (without 5-AZA) and a blank control (without cells) were included in each plate. At the end of treatment course, Cell Counting Kit-8 assay (Dojindo cat#CK04) was used to exam the cell proliferation status. The optical density (OD) was determined at 490 nm using an ELISA reader (BIORAD). Cell viability = (OD of treated-OD of brank)/(OD of the negative control-OD of the blank) × 100%.

As described above, four CRC cell lines and normal NCM460 cells were seeded in six-well plate and exposed to 5-AZA at a concentration of 5 μM for 48 h. The control group was treated in parallel with PBS. DNA, RNA and protein were harvested for downstream analysis. Each sample was tested in triplicate.

### Overexpression plasmid and siRNA transfection

Human vector pEnter-PCDH18 encoding the full-length open reading frame of the human PCDH18 gene and the control vector were purchased commercially (Vigene Biosciences Inc., cat#CH801462). All the vector sequences and orientations were confirmed by sequencing. CRC cell lines SW480 and SW620 were transfected with pEnter-PCDH18 or control plasmid using Lipofectamine 2000 (ThermoFisher Scientific Inc. cat#11668027). Stably transfected cells with PCDH18 expression were established under selection with kanamycin for 1 week. Through PCDH18 gene and protein expression detection, several positive clones were mixed for follow-up experiment. Cell Counting Kit-8 assay was used to exam the cell proliferation status.

PCDH18-siRNA (siPCDH18) and nontargeting negative control siRNA (siNC) (GenePharm) were transfected into normal human colorectal epithelial cell line NCM460 respectively using Lipofectamine 2000. The following sequences were used: PCDH18, sense: 5′-GGAGCCGAUAUGAAUUUGUTT-3′ and antisense: 5′-ACAAAUUCAUAUCGGCUCCTT-3′; negative control, sense: 5′-UUCUCCGAACGUGUCACGUTT-3′ and antisense: 5′-ACGUGACACGUUCGGAGAATT-3′. Each sample was tested in triplicate.

### DNA and RNA extraction

Histological evaluation of surgical specimens assured that all specimens studied consisted of at least 70% tumor cells^36^. Genomic DNA was isolated from cell lines and tissues using TIANamp Genomic DNA Kit (TIAGEN cat#DP304). Cell-free DNA was extracted from 1 ml plasma using QIAamp DNA Midi blood kit (Qiagen cat#51185). All procedures were strictly carried out according to the manufacturer’s instructions. The DNA was eluted in EB buffer, and stored at −80 °C. Total RNA was extracted from cell lines and tissues by Trizol reagent (Invitrogen cat#15596026). Extracted DNA and total RNA from cell lines and tissues was quantitated by NanoDrop 2000 (ThermoFisher Scientific Inc.). Plasma DNA was quantitated using Qubit dsDNA Assay Kit and Qubit 3.0 fluorometer (ThermoFisher Scientific Inc. cat#Q32851).

### Quantitative methylation-specific PCR (qMSP) and bisulfate sequencing PCR (BSP)

The extracted DNA was modified with sodium bisulfate to covert unmethylated cytosines to uracils using the EpiTech Bisulfite Kit (Qiagen cat#59104) according to the manufacturer’s protocol. Notably, the entire bisulfite converted plasma DNA was limited (6–15 ng/ml) and thus we used the EpiTect Whole Bisulfitome Kit (Qiagen cat#59203) to amplify the converted plasma DNA to obtain sufficient DNA for the downstream analysis. DNA methylation level of PCDH18 was analyzed using a SYBR Green based qMSP^34^. The primer sequences of PCDH18 were for the unmethylated reaction: 5′-GTGTTTTTTTTTTTGTGTAGTT-3′ (forward) and 5′-ACAAATATTTAATCTACAACCA-3′ (reverse) and for the methylated reaction: 5′- GCGTTTTTTTTTTTGTGTAGTC-3′ (forward) and 5′-ACGAATATTTAATCTACAACCG-3′ (reverse). EpiTect methylated DNA and unmethylated DNA (Qiagen cat#59695) were used as methylation and unmethylation positive control. In the PCR (10 μl), 1 μl of bisulfate-treated DNA template was mixed with 5 μl of 2 × SYBR *Premix Ex Taq* II (TAKARA cat#RR820A) and a pair of primers in a final concentration of 400 nmol/l. All the cycling conditions of PCR contained initial incubation at 50 °C for 2 min, denaturing at 95 °C for 5 min, followed by 40 cycles of 95 °C for 30 s, various annealing and extension temperatures (58 °C/PCDH18-M, 54 °C/PCDH18-U) for 1 min (ABI ViiA 7 Real-Time PCR System). After PCR amplification, a dissociation curve was generated to assess the quality of PCR product. Each sample was tested in duplicate.

For BSP analysis, the bisulfite-treated DNA of SW620 and NCM460 cell was amplified by PCR with the following primers: 5′-TTTTTTAGGTAGGAGAGTGTTATTTT-3′ (forward) and 5′-AAATTTTAAACTAAAATCAACAATCAC-3′ (reverse). PCR products were cloned into the pUC18-T vector, and five clones selected and sequenced from each sample.

### Quantitative PCR (qPCR) analysis of PCDH18 mRNA expression

The extracted total RNA was reverse-transcripted into cDNA using PrimeScript RT reagent Kit (TAKARA cat#RR047A) according to the manufacturer’s protocol. The relative PCDH18 mRNA expression was calculated using the comparative CT method, with GAPDH as a control. Primers used for PCDH18 were sense: 5′-AAGAATTCCCAACGCAACCC-3′ and anti-sense:5′-AATAGGTGTCCAGGGAAGGC-3′. Primers used for GAPDH were sense: 5′-TCAACGGATTTGGTCGTATTGGGC-3′ and anti-sense:5′-TCCTGGAAGATGGTGATGGGATTT-3′. The qPCR conditions consisted of initial incubation at 50 °C for 2 min, denaturing at 95 °C for 5 min, followed by 40 cycles of 95 °C for 30 s, 61 °C for 1 min (ABI ViiA 7 Real-Time PCR System). Each sample was testes in duplicate for expression.

### Western blot

Tissues and cells were lysed in cold RIPA buffer containing a proteinase inhibitor Phenylmethanesulfonyl fluoride (Beyotime cat#ST506) and a phosphatase inhibitor (Thermo scientific Inc. cat#78420). The protein concentrations were determined by the BCA protein assay (Thermo Scientific Inc. cat#23227). The samples were adjusted to equal protein concentrations and subjected to sodium dodecyl sulfate–polyacrylamide gel electrophoresis. Proteins on the gel were transferred onto PVDF membranes, and then were blocked with 5% bovine serum albumin in Tris-buffered saline containing 0.1% Tween 20 for 1 h at room temperature. The membranes were incubated with the anti-PCDH18 (Santa Cruz cat#sc-104574), anti-nonphospho (active)-β-catenin, anti-phospho-β-catenin, anti-β-catenin, anti-phospho-GSK-3β, anti-GSK-3β, anti-caspase3 (Cell Signaling Technology cat#8814, 4176, 8480, 9331, 9315, 9662), anti-p21, anti-cyclin A1, anti-cyclin D1, anti-cyclin E1 (Bioworld cat#AP0713, BS1084, BS2436, BS1085) and anti-GAPDH (Kangchen cat#KC5G4) overnight at 4 °C. After being washed with Tris-buffered saline containing 0.1% Tween 20, the membranes were incubated with the appropriate horseradish peroxidase-conjugated secondary antibody (ZSGB-BIO). The immunoreactive bands were detected with ECL kit (Lu long Inc. cat#ECL-100-ES) and cropped from Supplementary Fig. 2. Each sample was tested in duplicate.

### Immunohistochemical analysis of human CRC tissue microarray (TMA)

Human CRC TMA (Cat# HColA030PG03) was purchased from OUTDO BIOTECH (Shanghai, China). The TMA included adjacent normal tissues and matched CRC tissues from 14 individual patients. The TNM classification was provided in the Supplementary Table. S2. CRC tissue array slide was stained with primary antibody for PCDH18, followed by secondary antibody incubation, and was analyzed under a light microscope. The intensity of the staining signal was measured using the Image-Pro Plus 6.0 software (Media Cybernetics, Inc.). The signal density of tissue areas from three randomly selected visions were counted and subjected for statistical analysis. Each sample was tested in duplicate.

### Wound healing assays

All the cell lines were cultured in six-well plates until 70–80% confluent. The cell layers were carefully wounded using a sterile 20 μl tip and then washed twice with PBS. Cell culture medium contained a concentration of 0 (PBS) and 5 μM of 5-AZA, respectively. The wounds were photographed at 0 and 24 h. Each sample was tested in duplicate.

### Colony formation assays and transwell assays

Cells transfected with siRNA or overexpression plasmid were collected, re-plated and cultured for 2 weeks in complete growth medium at a density of 400 cells per well. Colonies were observed following staining with Crystal Violet Staining Solution.

Cell migration was also performed in triplicate using transwell assay. Two millicell inserts with 8 μM diameter pores were placed into a 24-well plate seeded with 1 × 10^4^ Cells transfected with siRNA or overexpression plasmid in serum-free medium, respectively. The lower chamber contained 500 μl medium with 10% FBS. Following 24 h of incubation, the cells on the upper membrane surface were scraped using a cotton swab and the migrated cells on the lower surface were fixed and stained with Crystal Violet Staining Solution.

### Cell cycle assay

Following 48 h of incubation, NCM460 cells (1 × 10^6^) transfected with siPCDH18 and siNC were harvested and wash twice wish PBS, fixed with ice-cold 70% ethanol overnight at 4 °C. Then, the cells were treated with 20 mg/l propidium iodide (PI) (Thermo Scientific Inc. cat#P1304MP) at 4 °C for 30 min in the dark and sorted by Beckman coulter cell Lab Quanta SC. The cell phase distribution was analysed by ModFit LT 3.1 software (Verity Software House, USA). Each sample was tested in duplicate.

### Immunofluorescence staining (IF)

Cell lines treated with 5-AZA at 0 (PBS) and 5 μM as well as NCM460 cells transfected with siPCDH18 and siNC were cultured onto small coverlips for 48 h, respectively. After washing three times with PBS, the cells were fixed with 4% paraformaldehyde for 20 min at 4 °C, then permeabilized for 4 min in 0.1% Triton X-100 in PBS. Cells were blocked for 1 h with 2% bovine serum albumin in PBS and then incubated with anti-PCDH18 and anti-phospho-β-catenin overnight at 4 °C. Nuclei were stained with DAPI (Thermo Scientific Inc. cat#D1306). Images were captured using a fluorescence microscope (OLYMPUS). Each sample was examined in duplicate.

### Imunoprecipitation (IP)

NCM460 cells transfected with siPCDH18 and siNC were washed with ice-cold PBS and the protein was lysed in cold RIPA buffer containing a proteinase inhibitor described above at 4 °C for 30 min. Extracts were centrifuged at 10000 rpm for 10 min at 4 °C, and the supernatant containing 1.0–1.5 mg protein/ml, was incubated with 2 μg of anti-PCDH18 at 4 °C overnight. Immune complexes were isolated by precipitation using protein G Agarose (for 1 h at 4 °C) (Merck Millpore cat#16-266). Washed beads were suspended in 30 μl of RIPA buffer and heated at 100 °C for 5 min. Extracts were immunoblotted for β-catenin and PCDH18 using Western blot.

### Statistical analysis

The percentage of PCDH18 methylation in a sample was estimated using the following formula: methylated PCDH18 (%) = 1/1 + 2^(−ΔCt)^ × 100%, where M is the copy number of methylated PCDH18, U is the copy number of unmethylated PCDH18, and Ct = Ct_U_ − Ct_M_^34, 37^. PCDH18 methylation status was treated as a continuous variable and a categorical variable. The differences in PCDH18 methylation status as a continuous variable by age, sex, tumor stage and Tumor location was calculated using t-test or rank sum test. Statistical analyses were carried out using software (GraphPad Prism for Windows, version 5.00; GraphPad Soft-ware Inc., La Jolla, CA). Receiver-operating characteristic (ROC) curves were calculated to evaluate the diagnostic performance of PCDH18. Molecular interaction network involving PCDH18 was analyzed and the map was generated by using Cytoscape 3.3.0 from different species^38^. Summary data was reported as mean ± SD. Group means were analyzed using the two-tailed Student’s t-test, where P < 0.05 was considered statistically significant.

## Electronic supplementary material

Supplementary Information
