# Supplementary material for: PCDH18 is frequently inactivated by promoter methylation in colorectal cancer
Source: Sci Rep. 2017 Jun 6;7:2819. doi: 10.1038/s41598-017-03133-w (PMC5460281; doi:10.1038/s41598-017-03133-w)
Supplement: Supplementary file 1 — Supplementary Information [file 41598_2017_3133_MOESM1_ESM.pdf]

PCDH18 is frequently inactivated by promoter methylation in colorectal cancer

Dan Zhou[1, 2, 3, 5], Weiwei Tang[1, 5], Guoqiang Su[4], Mingquan Cai[1], Han-Xiang An[1*] and Yun Zhang[1, 2, 3*]

[1]Department of Medical Oncology, The First Affiliated Hospital of Xiamen University, Xiamen, Fujian, China

[2]Xiamen Institute of Rare Earth Materials, Chinese Academy of Sciences, Xiamen, Fujian, China

[3]Key Laboratory of Design and Assembly of Functional Nanostructures, Fujian Provincial Key Laboratory of Nanomaterials, Fujian Institute of Research on the Structure of Matter, Chinese Academy of Sciences

[4]Department of Gastrointestinal surgery, The First Affiliated Hospital of Xiamen University, Xiamen, Fujian, China

[5]These authors contributed equally to this work.

[*]Corresponding author: Yun Zhang, Xiamen Institute of Rare Earth Materials, Chinese Academy of Sciences, 17th Floor, Technovation Build, Ji-mei Rd, Xiamen, Fujian, 361021, China; zhangy@fjirsm.ac.cn

Han-Xiang An, Department of Medical Oncology, The First Affiliated Hospital of Xiamen University, Xiamen, 361003, Fujian, China; anhanxiang@xmu.edu.cn

Supplementary Table. S1 Clinical characteristics of patients with colorectal cancer and normal controls

Clinicopathologic factors	Tumor tissue study				Plasma study	
	qMSP/qPCR/Western blot		Immunohistochemistry		qMSP	
	CRC Cases	Adjacent normal tissue	CRC Cases	Adjacent normal tissue	CRC Cases	Healthy controls
**Total**	29	29	14	14	20	20
**Mean Age ±SD**	60.3 ±14.7		61.0 ±7.4		55.8 ±14.7	51.4 ±12.8
**Sex**						
Male	17		9		10	11
Female	12		5		10	9
**T stage**						
T1-T2	7		0		2	
T3-T4	19		0		16	
NA	3		14		2	
**N stage**						
N0-N1	21		11		11	
N2-N3	5		3		7	
NA	3		0		2	
**M stage**						
M0	19		14		15	
M1	7		0		3	
NA	3		0		2	
**stage**						
I	3		0		1	
II	8		14		4	
III	8		0		9	
IV	8		0		3	
NA	2		0		3	
**Tumor location**						
Proximal colon (cecum to transverse)	7		8		4	
Distal colon (splenic flexure to sigmoid)	8		6		5	
Rectum	14		0		11	

CRC, colorectal cancer. qMSP, quantitative methylation specific PCR. NA, not available.

Supplementary Table. S2 Clinical characteristics of patients with colorectal cancer and normal controls in Tissue Array

Lot No.	HColA030PG03
CGt No.	XT16-005
Product description	Colorectal cancer
Sample type	Human
Fixed mode	Formalin
Number	14 patients
Diameter	2.0mm

The distribution of the tissue array

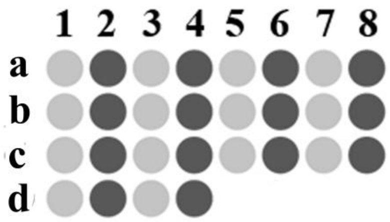

Location	No	Tissue Type	Histological type	Stage	Comment
A1	D15A0691-B30-C1	Cancer	Colon adenocarcinoma	II	
A2	D15A0691-B30-P1	Normal	Colonic mucosa		
A3	D15A0692-B30-C1	Cancer	Colon adenocarcinoma	II	
A4	D15A0692-B30-P1	Normal	Colonic mucosa		
A5	D15A0695-B30-C1	Cancer	Colon adenocarcinoma	II	
A6	D15A0695-B30-P1	Normal	Colonic mucosa		
A7	D15A0696-B30-C1	Cancer	Colon adenocarcinoma	II	
A8	D15A0696-B30-P1	Normal	Colonic mucosa		
B1	D15A0697-B30-C1	Cancer	Colon adenocarcinoma	II	
B2	D15A0697-B30-P1	Normal	Colonic mucosa		
B3	D15A0698-B30-C1	Cancer	Colon adenocarcinoma	II	
B4	D15A0698-B30-P1	Normal	Colonic mucosa		
B5	D15A0699-B30-C1	Cancer	Colon adenocarcinoma	II	

B6	D15A0699-B30-P1	Normal	Colonic mucosa		
B7	D15A0700-B30-C1	Cancer	Colon adenocarcinoma	II	
B8	D15A0700-B30-P1	Normal	Colonic mucosa		
C1	D15A0701-B30-C1	Cancer	Colon adenocarcinoma	II	
C2	D15A0701-B30-P1	Normal	Colonic mucosa		
C3	D15A0703-B30-C1	Cancer	Colon adenocarcinoma	II	
C4	D15A0703-B30-P1	Normal	Colonic mucosa		
C5	D15A0704-B30-C1	Cancer	Colon adenocarcinoma	II	
C6	D15A0704-B30-P1	Normal	Colonic mucosa		
C7	D15A0707-B30-C1	Cancer	Colon adenocarcinoma	II	
C8	D15A0707-B30-P1	Normal	Colonic mucosa		
D1	D15A0708-B30-C1	Cancer	Colon adenocarcinoma	II	
D2	D15A0708-B30-P1	Normal	Colonic mucosa		
D3	D15A0709-B30-C1	Cancer	Colon adenocarcinoma	II	
D4	D15A0709-B30-P1	Normal	Colonic mucosa		

A CTTTCCAGGCAGGAGAGTGTCACCTC**CGCG**TTTTCTCCCTTGTGTAGT**CG**GA
ATCCATCTATTGCAGGGTGC**CG**GTGGAGTCTGCAGTGTGTCTTTCCTGAG**CG**
ATTCTTTCTCCCTTTAGCTGCT**CG**GCTGCAGACTAAACACC**CG**TGATTGCTGA
CTCCAGTCTAAAATCT

B

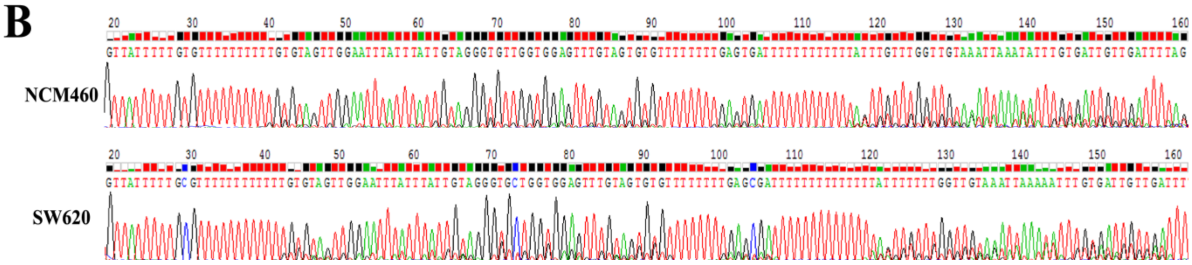

Supplementary Fig. 1 (A) Illustration of the amplified fragment in PCDH18 promoter for bisulfate sequencing PCR (BSP). The red bold "CG" indicates the location of CpG island sites. Black rectangles indicate the primers for BSP. (B) Representative results of BSP. The blue "C" indicates methylated CpGs.

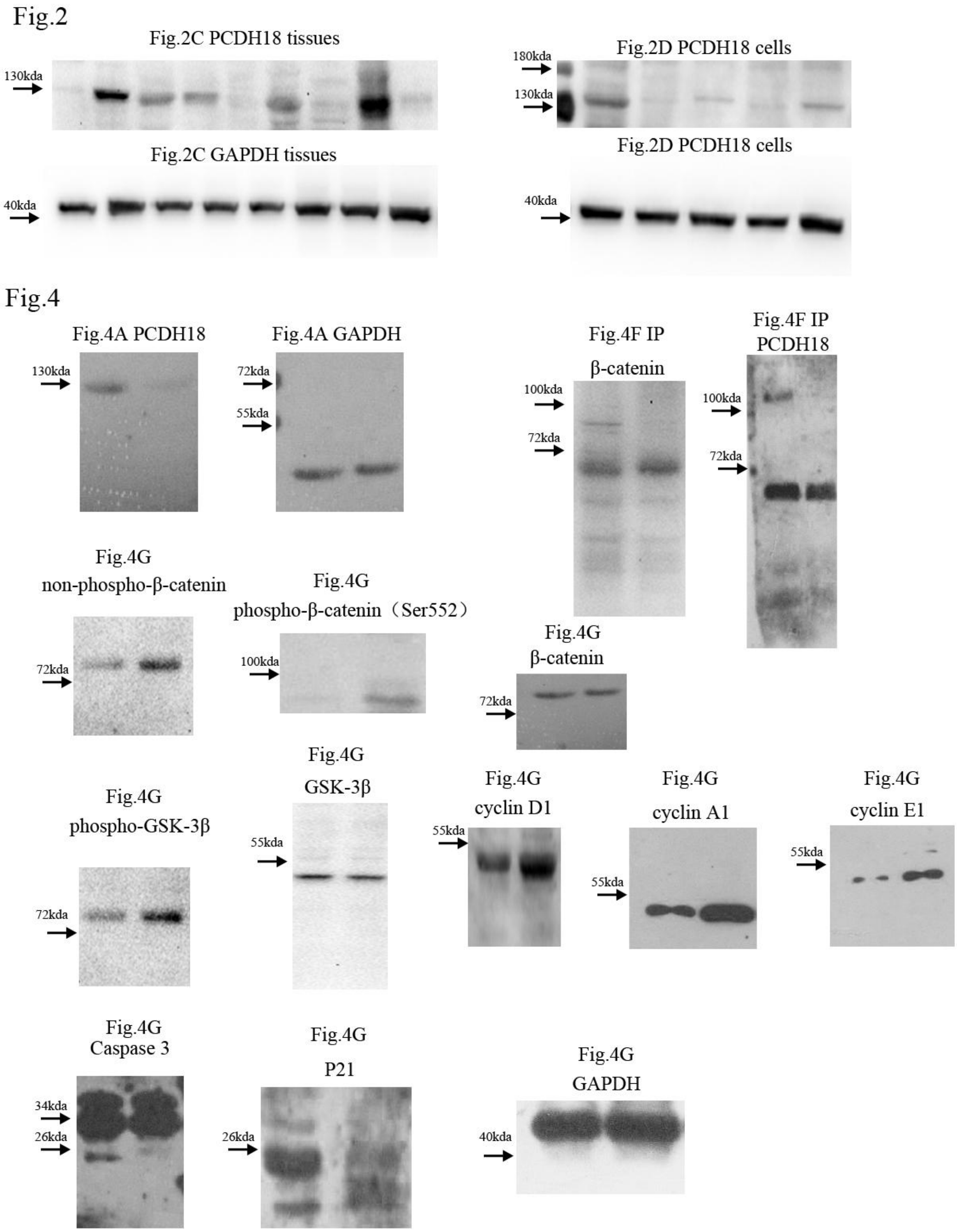

Supplementary Fig. 2 Full length Western blot images.
